# Beyond Glioma: The Utility of Radiomic Analysis for Non-Glial Intracranial Tumors

**DOI:** 10.3390/cancers14030836

**Published:** 2022-02-07

**Authors:** Darius Kalasauskas, Michael Kosterhon, Naureen Keric, Oliver Korczynski, Andrea Kronfeld, Florian Ringel, Ahmed Othman, Marc A. Brockmann

**Affiliations:** 1 Department of Neurosurgery, University Medical Centre, Johannes Gutenberg University Mainz, 55131 Mainz, Germany; Darius.Kalasauskas@unimedizin-mainz.de (D.K.); mikoster@uni-mainz.de (M.K.); Naureen.Keric@unimedizin-mainz.de (N.K.); Florian.Ringel@unimedizin-mainz.de (F.R.); 2 Department of Neuroradiology, University Medical Centre, Johannes Gutenberg University Mainz, 55131 Mainz, Germany; oliver.korczynski@unimedizin-mainz.de (O.K.); Andrea.Kronfeld@unimedizin-mainz.de (A.K.); ahmed.othman@unimedizin-mainz.de (A.O.)

**Keywords:** radiomics, CNS tumor, metastasis, meningioma, pituitary, CNS lymphoma, medulloblastoma, schwannoma

## Abstract

**Simple Summary:**

Tumor qualities, such as growth rate, firmness, and intrusion into healthy tissue, can be very important for operation planning and further treatment. Radiomics is a promising new method that allows the determination of some of these qualities on images performed before surgery. In this article, we provide a review of the use of radiomics in various tumors of the central nervous system, such as metastases, lymphoma, meningioma, medulloblastoma, and pituitary tumors.

**Abstract:**

The field of radiomics is rapidly expanding and gaining a valuable role in neuro-oncology. The possibilities related to the use of radiomic analysis, such as distinguishing types of malignancies, predicting tumor grade, determining the presence of particular molecular markers, consistency, therapy response, and prognosis, can considerably influence decision-making in medicine in the near future. Even though the main focus of radiomic analyses has been on glial CNS tumors, studies on other intracranial tumors have shown encouraging results. Therefore, as the main focus of this review, we performed an analysis of publications on PubMed and Web of Science databases, focusing on radiomics in CNS metastases, lymphoma, meningioma, medulloblastoma, and pituitary tumors.

## 1. Introduction

The radiological assessment of pathological changes in medical imaging is usually done by a qualitative analysis of a limited number of categories (i.e., size, appearance, and lesion borders). However, imaging data is affluent in information, which may be additionally extracted quantitatively using various radiomic methods. Particularly in neuro-oncology, radiomics can be implemented for tumor identification, characterization, and response prediction [1]. 

Radiomics, a potential tool for the diagnosis and treatment of central nervous system (CNS) diseases, has evolved in recent years with the ongoing growth of publications. The majority of radiomic analyses have been conducted on glial CNS tumors, revealing the ability of radiomics to characterize tumor aggressiveness, mutation, and response [2,3]. Nevertheless, gliomas account for only one-eighth of CNS tumors [4]. The assessment of radiomics in other intracranial tumors may also impact their identification, characterization, and response prediction and positively affect patient care. Some researchers have explored radiomic analyses on various brain tumor types (primary and secondary brain tumors) other than gliomas, with promising results. The findings of these studies may benefit from a summarization, making them easily accessible for health care professionals and researchers. Therefore, to reinforce its importance and provide a comprehensive overview of this topic, we reviewed radiomic analyses of non-glial brain tumors containing all common primary and secondary tumor entities. After a short description of the applied search methods, the identified studies will be discussed regarding their study design and relevant findings. This review will encompass radiomic studies on brain metastases (BMs), the most common brain tumors. We will also discuss studies on CNS lymphomas, a relatively rare disease with a poor prognosis, and focus on the most common malignant pediatric tumors (medulloblastomas (MBs)), a heterogeneous group of tumors with different molecular characteristics and outcomes. We will explore benign meningiomas in the context of radiomics, focusing on grading and morphological overlap with other tumor entities such as solitary fibrous tumors. We will also evaluate the ability of radiomic analysis for characterization and response prediction of pituitary adenomas, as well as give a brief overview of radiomic research related to other less common CNS tumors. The main results will then be summarized in the Table S1 which is aimed to serve as a quick reference tool.

## 2. Methods

**Figure 1:**
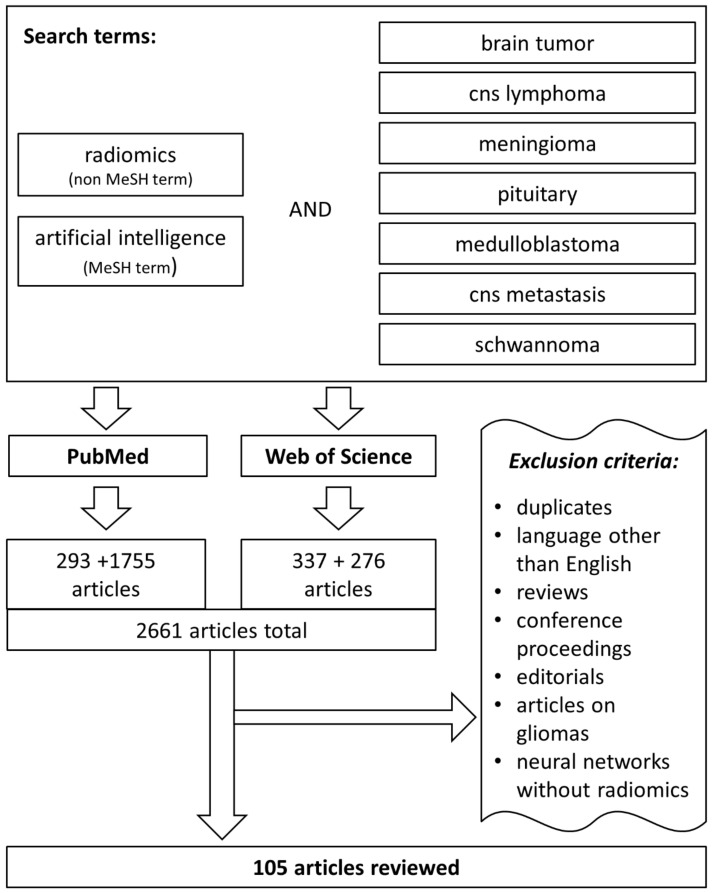
The representation of the article selection process.

We conducted a search in PubMed and Web of Science databases on April 20th, 2021, starting with 1990 and using the keyword “radiomics” combined with one of the following keywords: “brain tumor”, “CNS lymphoma”, “central nervous system lymphoma”, “meningioma”, “pituitary”, “medulloblastoma”, “CNS metastasis”, “central nervous system metastasis”, or “schwannoma”. The search generated 293 and 337 results, respectively. Since “radiomics” is not a MeSH term, the search was expanded using the keyword “artificial intelligence” instead of radiomics. This search provided 1755 and 276 results, respectively. Articles on glioma, abstracts, conference proceedings, reviews, editorials, and articles in languages other than English were omitted. In addition, we focused on the use of predefined (handcrafted) radiomic features; therefore, numerous studies dealing with the use of deep-learning algorithms and automated features were excluded. These measures reduced the number of articles to 105. The article selection process is depicted in Figure 1. 

**Figure 2:**
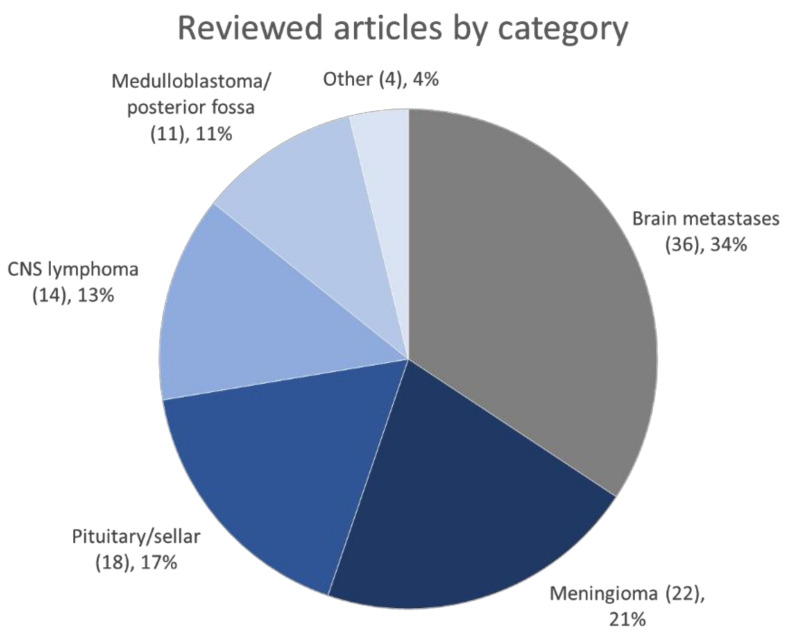
Number of reviewed articles by category.

We aimed to provide the reader with a summary of the studies reviewed after the selection process, including the study objective, number of patients, number of extracted features, methods of feature selection, and model building. The summary is provided in Table S1. Where possible, the area under the curve (AUC) or the accuracy of the model was recorded. If possible, the conservative value was extracted from the validation (ideally, external validation) dataset. The Table S1 should serve solely as an orientation and quick reference tool. For detailed information, refer to the original articles. Figure 2 displays the number of reviewed articles sorted by category.

## 3. Metastases

Approximately one-half of diagnosed brain tumors are metastases from other malignancies [5]. Diagnosis of these lesions and differentiation between different types of metastases often remains challenging due to their similarity in MRI to other primary brain tumors, such as glioblastoma (GBM). Moreover, the differentiation of radionecrosis and tumor progression after radiation can be difficult. Additional workup in such cases may include positron emission tomography (PET) imaging [6]. The differentiation between various types of metastases can be a difficult task, especially in patients with a history of multiple carcinomas or when the primary site of the malignancy is not known, as in cancers of unknown primary (CUP). 

Moreover, targeted tumor therapy based on identifying sensitive genetic mutations is gaining interest in the treatment of many tumor entities. As the mutation status of metastases can vary from the primary lesion [7], radiomics might also be a valuable tool to correlate different gene expression patterns to imaging parameters. Another potential of radiomics might lie in predicting patient outcomes after radiation or chemotherapy. These findings might allow better patient stratification and further guide treatment selection. The publications dealing with radiomics and BM, depending on the objective of the radiomic analysis, are summarized based on the category of study objective.

### 3.1. Differentiation between Brain Metastases and Other Entities

Due to the visual similarities of primary brain tumors and BM, the discrimination of these entities on MRI conventional methods can be challenging. Particularly in newly diagnosed tumors of unknown primary, the differentiation between BM and primary brain tumors is notional unless histological confirmation is available. Radiomics may have the potential to improve the accuracy of tumor discrimination further.

A total of 11 studies were identified. The majority of the studies utilized at least contrast-enhanced T1 (CE T1) images for feature extraction [8,9,10,11,12,13,14,15,16], but many also used additional standard sequences such as T1, T2, contrast-enhanced T2, T2 gradient echo, ADC, and FLAIR. Sartoretti et al. used amide proton-transfer weighted (APTw) imaging and found a sensitivity of 81.3% and a specificity of 81.1% to distinguish between primary brain tumors and metastases [17]. Diffusion tensor imaging-derived features have been used with great accuracy [15,18,19]. Another group used MRI fingerprinting, a newly developed technology measuring the signal progress within every voxel during acquisition [20]. This technique achieved the most accurate separation of GBMs and metastases using T1 sequences, with AUC reaching 0.877 [20]. Two studies with the largest patient cohorts (439 and 412 patients) [8,10] utilized 757/1303 radiomic features extracted from contrast-enhanced T1 (and additional T1 and T2 in [8]). Support vector machines were used as machine-learning classifiers and yielded AUCs of 0.96 and 0.9 for differentiation between GBM and BM, respectively. The model-building method yielding the best performance is not readily identifiable. Neural networks can successfully be used with or without the extraction of handcrafted radiomic features for tumor classification [16,21]. The possibility to choose between several machine-learning methods can enhance results even in limited patient populations [13,18]. Moreover, the clinical performance of the best classifier was superior to that of a neuroradiologist in accuracy, sensitivity, and specificity. 

### 3.2. Differentiation between Radionecrosis and Tumor Progression 

Another challenge is the distinction between progression and pseudo-progression in patients after radiotherapy. We found seven publications on this topic; the number of patients varied from 20 to 160, and radiomics were primarily extracted from CE T1 sequences (in two studies, from additional sequences) [22,23,24]. AUC ranged between 0.73 and 0.83. One group focused on local anisotropic gradient orientations (COLLAGE) co-occurrence, a recently developed radiomic feature that captures entropy [25]. The feature was extracted from CE T1, cerebral radiation necrosis, and recurrent tumors could be differentiated using the Wilcoxon rank sum test.

Several studies were focused on extracting radiomic features from PET scans (some in combination with MRI). Lohmann et al. found only a slight improvement when using the standard FET-PET parameters TBRmean and TBRmax (AUCs 0.81 and 0.83) alone or in combination with radiomic features (AUCs of 0.85 for both) [26]. The same workgroup also compared radiomics of CE T1 and FET-PET and found AUCs of 0.91 for FET-PET alone vs. 0.85 for MRI (CE T1) alone. Combined with both imaging modalities, the precision raised to an AUC of 0.96 [27]. Finally, Hotta et al. investigated 11-C-methionine-PET and compared radiomic feature analysis with the accuracy of the classic PET metric tumor-to-normal cortex (T/N) ratio [28]. A random forest classifier, together with a ten-fold cross-validation, was used to analyze a total of 42 radiomic features. Exact differentiating progression from radionecrosis in GBM patients with an AUC of 0.73 for T/N versus an astonishing AUC of 0.98 for radiomic features was reported. 

### 3.3. Differentiation between Different Types of Metastasis

We found four studies based on radiomics in MRI to differentiate between different metastatic types. The largest included 189 patients with 658 BMs (breast cancer *n* = 143, small-cell lung cancer *n* = 151, non-small-cell lung cancer *n* = 225, gastrointestinal cancer *n* = 50, melanoma *n* = 89) [29]; 1423 radiomic features were extracted from T1, CE T1, and FLAIR and fed into a random forest classifier. The AUCs ranged from 0.64 (for non-small-cell lung cancer) to 0.82 (for melanoma) and were insufficient for routine diagnostics. However, the radiomics analysis outperformed the radiologists’ readings in all cases. Another interesting study was intended to investigate the differentiability of lung cancer, breast cancer, and melanoma metastases using 2D and 3D features with different gray-level quantizations (8, 16, 32, 64, 128) and a random forest classifier in CE T1 images [30]. A mean AUC of 0.87 was reported for the top four features out of 43 in an optimal dataset (3D, 32 gray levels). The AUCs for differentiating lung from breast and lung from melanoma metastases were 0.96 and 0.94, respectively. 

On the other hand, the classification of breast cancer and melanoma BMs was unsatisfactory (AUC = 0.607). In a similar study, researchers implemented a naïve Bayes classifier and reported an AUC of 0.95 [31]. Moreover, the use of CE CT imaging-based radiomics to differentiate adenocarcinoma from squamous cell cancer in lung cancer patients with BMs was also reported. Finally, a promising AUC of 0.83 was reported using binary logistic regression paired with clinical data as a classifier [32].

### 3.4. Prediction of Mutation Status in Metastasis 

We found five studies in which radiomics were used to differentiate BMs by their mutation status. Four of them were intended to identify the EGFR mutation status in NSCLC BMs using (at least) CE T1 images [33,34,35,36]. The AUCs varied from 0.71 to 0.991. The most extensive study by Chen et al. included 110 patients and investigated EGFR, ALK, and KRAS mutation status [37]. A list of 2786 features was reduced to 50 significant features and supplemented by clinical data. Satisfactory accuracy for the prediction of EGFR (AUC 0.91), ALK (0.92), and KRAS (AUC 0.99) was achieved using a random forest classifier. One researcher investigated the BRAF mutation status in melanoma BMs [38]. A linear support vector machine classifier yielded an AUC of 0.78 using 50 out of 195 radiomic features from CE T1 paired with clinical data.

### 3.5. Prediction of Outcome in Response to Radiation or Chemotherapy

We found seven articles on the ability of radiomics to predict responses to radiation therapy [37,39,40,41,42,43,44]. One study was intended to investigate outcome predictions after applying checkpoint inhibitors in melanoma patients using radiomics [45]. In the mentioned studies, 48–161 patients were investigated, mainly using radiomic features extracted from CE T1 images—in one case, from PET-examinations (11-C-MET-PET) [40], and in another case, from CE CT [42]. AUCs of 0.73 based on PET and AUCs of up to 0.86 based on CE CT were found for differentiation between responders and non-responders after stereotactic radiosurgery (SRS). Chen et al. investigated overall survival time after SRS and demonstrated high diagnostic accuracy for predicting overall survival [37]. Kawahara et al. used radiomics from FLASH T1 sequences of 157 melanoma patients with BM to predict response to SRS. They yielded an accuracy of 88% in an NN classifier compared to only 44% by visual inspection [39]. Two similar publications using cohorts with BMs of miscellaneous origin (87 and 110 patients) reported AUCs of 0.79 and 0.79, respectively, to predict SRS response [41,43]. CE T1 and FLAIR were used in both studies. 

Moreover, the prediction of responses to checkpoint inhibitor therapy in melanoma patients with BMs was investigated [45]. A total of 88 patients with 196 BMs received either ipilimumab (72%), programmed cell death protein one blockade (13%), or nivolumab plus ipilimumab (16%). Twenty-one radiomic features were extracted from CE T1 images, and multivariate analysis was performed. Multiple radiomic features were associated with patient survival. The authors concluded that higher-order MRI radiomic features in patients with melanoma BMs receiving checkpoint inhibitors were associated with a trend toward improved overall survival.

BM is currently the largest category in publications concerning radiomics in non-glial CNS tumors. The majority of papers we reviewed investigated differentiation of BM from primary brain tumors, pseudoprogression, and prediction of response to radiation therapy. T1 CE, T1, and FLAIR sequences were most commonly used. The differentiation between types of metastasis is accurate, though the reliability of differentiating mutation status within BMs varies a lot among different BM types. Nevertheless, the high accuracy of radiomic methods might be a viable alternative for currently established diagnostic procedures, such as tumor biopsy or PET scans, in the future.

## 4. Primary CNS Lymphoma

Much effort was made to differentiate GBM and lymphoma preoperatively, as these two tumor entities may have a similar appearance on MRI. In total, 13 studies that we reviewed addressed the differentiation between lymphoma and GBM and 1 the differentiation between neurosarcoidosis and lymphoma. Preoperative determination of tumor entity is essential, as the treatment strategies are entirely different.

Many studies have shown a remarkable potential of radiomics for the differentiation of lymphoma and GBM, with the AUC exceeding 0.97 in certain cases [46,47,48,49,50,51,52,53]. The feature selection and model-building methods vary significantly among studies (see Table S1), and in some cases, the models include clinical or semantic variables. MRI sequences used for tumor differentiation vary as well and have, for example, CE T1 sequences only [46,52,54] as well as combinations of ADC and T1CE [55]; ADC, FLAIR, and CE T1 [47]; DWI, CE T1, and FLAIR [48]; CE T1, T2 and DWI [50]; T1, T2, and FLAIR [51,56]; or ADC and gradient-echo [15]. A study involving 141 patients reported over 90% accuracy in differentiation between various tumor entities, including GBM, lymphoma, BM, and meningioma, using five MRI sequences in total [15]. Moreover, in another study, the utility of radiomics in differentiation between BM and neurosarcoidosis was explored [57]. The authors could identify two features that enabled the discrimination between these two entities with a very high accuracy. Alternatively to the conventional approach of tumor volumetry, a representative axial slice can be used to draw a region of interest and differentiate between GBM and lymphoma with high accuracy [56]. Despite the considerable heterogeneity of these approaches, it seems that radiomics is a promising tool for the differentiation of lymphoma and GBM, as evidenced by a satisfactory AUC in some of these models. 

When dealing with primary CNS lymphoma, one common problem is that this is a rare tumor entity, making it challenging to generate a large dataset. Using various cross-validation techniques, the problem can be addressed using various classification strategies, including radiomic features and supervised machine learning or multilayer perceptron networks [58] using multiple cross-validation techniques. The synthetic minority oversampling technique (SMOTE) is used to balance imbalanced datasets to overcome overly optimistic classification estimates [59] in certain studies. In most cases, the radiomic approach is superior to classification by expert radiologists. For example, a combination of radiomic features, a multilayer perceptron network, and a neural network approach in selection and generalization was superior to radiologists’ expertise [58]. 

Besides conventional radiomic analysis, there are alternative approaches for tumor discrimination. The time-intensity curve analysis of enhancing tumor vs. normal-appearing white matter was utilized to discriminate lymphoma from other CNS tumors, with AUC reaching over 0.95 vs. GBM and lymphoma and 0.83 vs. metastases [60]. Based on DWI and DSC perfusion imaging, a multiparametric model had extraordinary accuracy using selective mean and maximum ADC, mean and maximum CBV, and ratio CBV [61]. As ADC values reflect high cellularity and a high nuclear/cytoplasmic ratio in CNS lymphoma, an ADC-based approach is promising for differentiating lymphoma and GBM. The accuracy in an external validation set demonstrating an AUC of 0.944 might even be superior to conventional radiomics or human readers [62]. However, according to another study, radiomics outperformed an ADC map and human reader-based classification [51]. Moreover, ^18^F-fluorodeoxyglucose (FDG) PET imaging can also be utilized for radiomic analysis. A standardized uptake value (SUV) map, an SUV map calibrated with the normal contralateral cortex (NCC) activity (SUV/NCC map), and an SUV map calibrated with normal brain mean (NBM) activity (SUV/NBM map) were used for radiomic analysis and demonstrated an AUC over 0.9 for discrimination between GBM and lymphoma concerning certain features [49]. 

Regardless of the chosen MRI sequences, features, selection methods, or statistical models, radiomics could outperform human readers in differentiating between GBM and CNS lymphoma. 

## 5. Medulloblastoma and Other Tumors of the Posterior Fossa

Preoperative differentiation of tumors of the posterior fossa and MB molecular subtypes could be helpful for surgical treatment planning. A relatively low prevalence and the specifics of pediatric populations meant that only 11 studies were identified during the search. Radiomic-based discrimination between adult and pediatric tumor entities (ependymoma, pilocytic astrocytoma, and MBs) have been done with promising accuracy and AUCs of over 0.9 in certain studies [63,64,65,66,67,68]. T1CE sequences and ADC maps were used in most of the models. However, several studies did not employ CE sequences and relied on T1, T2 or ADC maps [65,66,67]. Moreover, some authors were able to simplify the classification process by reducing the necessary features to three or five [63,68], thus, providing radiological biomarkers for the discrimination process. The radiomic approach seems promising for differential diagnosis of common tumors, such as MB, metastasis, and hemangioblastoma, a model that achieves an accuracy of 0.8 for tumor differentiation [69]. The radiomic-based classification of MB subgroups was more difficult. For example, a study of 122 patients demonstrated an AUC of 0.82 for WNT but much lower AUCs for other subgroups [70]. After adding age, gender, location, and hydrocephalus status, the accuracy increased to AUCs between 0.70 and 0.91 [70]. In another study, AUCs reached 0.70–0.79 for the sonic hedgehog subgroup and 0.8–0.83 for group 4, depending on the validation method [71]. The classification was less accurate for WNT and group 3 tumors. As for other tumor entities, radiomics signatures were identified as independent predictors of overall and progression-free survival [72].

Other investigated topics included incorporating clinical and radiomic features for predicting cerebral spinal fluid (CSF) dissemination in MBs with an AUC of 0.73 in an external validation cohort [73]. The radiomic approach was investigated as a possible predictor of treatment response after experimental intraventricular treatment [74]. However, results remained inconclusive due to a minimal number of patients (seven). 

## 6. Meningioma

Even though meningiomas are usually slow-growing, benign tumors, preoperative prediction of WHO grade or prognosis without histological analysis may be helpful for treatment planning. In total, 22 articles that met our inclusion criteria were reviewed. The majority of studies investigated the differentiation of histopathological grading [59,75,76,77,78,79,80,81,82]. CE T1 sequences were used in most of the studies; however, the best prognostic value (AUC over 0.95) was reached when other sequences, such as T2 and FLAIR, were included. In some studies, a combination of radiomic and other criteria (e.g., semantic radiological analysis) was used [78,83]. The radiomic approach can be helpful for preoperative differentiation of meningioma and other tumors. For example, differentiation between solitary fibrous tumors and angiomatous meningiomas was achieved with an AUC of over 0.90 [84,85,86]. Another study that was intended to differentiate common tumors of the anterior fossa using clinical; and radiomic features (pituitary adenoma, craniopharyngioma, meningioma, and Rathke cleft cyst) demonstrated a diagnostic performance of more than 0.80 in each group [87,88]. The same approach in the sellar region to differentiate between craniopharyngioma and meningioma demonstrated an AUC above 0.7 [89]. The utility of radiomic analysis for the identification of histological tumor subtype [90] or prognosis (tumor aggressiveness) [77,91] is promising. For example, the accuracy of identifying the meningioma subtype was over 90% [90]. In addition, integrated radiological, radiomic, and clinical models demonstrated AUCs for tumor relapse and overall survival of 0.75 and 0.78, respectively [77]. 

Some other features of meningiomas, which could be relevant for surgical planning, might be determined using radiomic analysis. For example, the consistency of meningioma (hard or soft) could be predicted using machine-based learning radiomic analysis with an AUC of 0.87–0.96 [92,93]. Prediction of postoperative cerebral edema could be achieved using machine-based learning radiomic analysis and achieved an AUC of 0.8 in a validation cohort [94]. Prediction of brain invasion could be determined with variable accuracy (AUC 0.71–0.91) in most studies using T1 CE and T2 sequences [95,96,97,98]. Some models were based on combined radiomic and radiologic predictors. However, the value of radiomics for predicting bone invasion is currently not optimal, with AUCs reaching 0.72 in one identified study [99]. Moreover, the aim to establish a prediction model for a dural tail infiltration using the radiomic approach was unsuccessful, mainly due to the thin layer of the dural tail and a small dataset [100]. 

Some alternative approaches have been investigated, as well. Fractal features, the measurement of geometrical complexity, and tumor lacunarity were used to predict WHO grade, which succeeded with an AUC of 0.81 [101]. ADC maps based on radiomic features, combined with clinical features, could predict WHO grade [79]. On the other hand, ADC mapping could predict meningioma relapse and histological grade [102]. 

Currently, the high predictive value of radiomics for meningiomas can be identified in the classification of WHO grade and differentiation between meningiomas and solitary fibrous tumors. Surgical qualities such as infiltration of surrounding tissue and tumor prognosis still need to be optimized, possibly by integrating more MRI sequences or using larger patient cohorts. 

## 7. Pituitary/Sellar Region Tumors

Due to the wide variety of tumors of the sellar regions, the studies identified during the literature review were heterogeneous; 18 articles were found. There were several studies aimed at identifying surgical qualities of tumors, such as prediction of tumor texture (hard vs. soft) [103,104,105] and invasion of the cavernous sinus [106]. These qualities could be predicted with a variable AUC between 0.8 and 0.99. Interestingly, the highest accuracy was reported in a study using a single T2 sequence for radiomic analysis [105]. Radiomic analysis of dynamic contrast-enhancement sequencing is promising in determining pituitary adenomas’ vascular heterogeneity and aggressiveness [107]. Several researchers have investigated differentiation between tumor entities and histological subtypes of pituitary tumors. The subtype prediction based on T2 weighted-image analysis reached an AUC over 0.95 [108]. The differentiation of nonfunctioning pituitary adenomas between null cell adenomas and other subtypes was not that accurate, with an AUC of 0.8 for the validation set [109]. 

Interestingly, contrast-enhanced sequences provided no additional contribution to the predictive model. Differentiation between pituitary adenomas, meningiomas, craniopharyngiomas, or Rathke cleft cysts was achieved with an AUC of 0.80 [87]. Several studies on histopathological subtypes of craniopharyngioma were also conducted; a model demonstrated an AUC of 0.89–0.92 for differentiation between adamantinomatous and papillary craniopharyngiomas, an AUC of 0.91 for BRAF V600E mutation, and AUC 0.93 for CTNNB1 mutation [110,111].

Another interesting application of radiomics in pituitary tumors might be the prediction of response rates to dopamine agonists in the treatment of prolactinomas. This prediction reached an AUC of 0.81 in the test set [112]. Histological qualities of pituitary adenomas were also investigated. Two studies showed promising results for predicting the Ki-67 labeling index using radiomic criteria [113,114]. Growth hormone-secreting adenomas granulation pattern was determined with an AUC of 0.83 [115]. According to four studies, treatment response could be predicted with an AUC of 0.8 or higher: in a model of therapeutic response in patients with functioning pituitary adenomas [116,117] and for progression or recurrence of non-functioning pituitary adenomas [118,119].

## 8. Other Tumors

After gamma knife surgery, a study on vestibular schwannomas focused on pseudoprogression and involved non-operatively managed patients. A two-level model demonstrated an accuracy of 85% in predicting pseudoprogression based on inhomogeneous hypointensity patterns of contrast enhancement and the variation of T2-weighted intensity, reaching 88% accuracy on long-term outcomes [120]. Another study showed promising results in predicting long-term tumor control after stereotactic radiosurgery of vestibular schwannomas [121]. Association with histological features such as mucin, lymphocytes, hemosiderin, and cellularity was identified [122], as well as promising classification of vestibular schwannomas into low and high blood supply [123]. Another study was intended to predict early radiation-induced temporal lobe injuries in nasopharyngeal carcinoma patients [124], with the best model achieving an AUC of 0.83. 

## 9. Limitations and Future Directions

In this review, we attempted to provide readers with a comprehensive, up-to-date reference on radiomics of non-glial tumors and encourage further research on radiomics. The studies analyzed in this review, including the aim, accuracy, and statistical approach are summarized in Table S1. The significance of this literature review relies mainly on the validity of the included studies. Therefore, this review has clear limitations. It was intended to be a narrative review with an extensive literature search. A formal systematic review, including deep-learning-based methods, or a formal meta-analysis on this topic may be an appropriate next step to explore the further potential of imaging analysis for non-glial brain tumors. Much research needs to be done concerning most of the included tumor entities, and practical everyday use of radiomic analysis still needs to be defined. Future research may prepare more profound reviews on various brain tumor entities. 

Despite the presented capabilities of radiomics in differentiating and prognosticating neuro-oncologic imaging, more evidence with regard to reproducibility and generalizability of radiomic features in neuro-oncology is needed. The reproducibility of radiomic features is limited by the extent of intra-individual test–retest repeatability, sensitivity toward the use of scanner types [125,126], and heterogeneous acquisition parameters and reconstruction algorithms [127,128]. Many of the studies discussed derive their findings from small samples. High-dimensional radiomic analyses yield a large number of features. Therefore, a sufficient assessment of reproducibility measures and data normalization may be required in radiomic research prior to external validation and eventual clinical application. Further standardization of segmentation (e.g., automated segmentation) and feature extraction methods is also encouraged to enhance the reproducibility of radiomic findings in general and in neuro-oncology in particular [129,130]. The use of numerous radiomic features as predictors in small samples is associated with severe overfitting and false-positive findings [131,132]. Many extracted features contain noise or are highly correlated with each other, making feature selection crucial to increase model accuracy. Stability tests, including scan–rescan and annotate–reannotate studies, can be done inside experiments on radiomics and could further improve repeatability and reproducibility [133]. Such studies may detect and filter unstable features. However, unstable features may be more predictive than stable features; thus, such filtering methods risk losing relevant predictive information. Götz et al. proposed a novel method of data augmentation for information transfer (DAFIT) that preserves unstable features and incorporates the results of side experiments into the predictive models, possibly enhancing future radiomics models [134]. Furthermore, feature selection performed via unsupervised methods such as principal component analysis may be more suitable, as they may reduce model overfitting [135]. Other methods for dimension reduction, such as random projection algorithms, have been proposed and could be suitable for reducing vast numbers of features. Internal validation, e.g., using sample splitting or cross-validation, is of particular importance for estimating model robustness. Finally, for the general implementation of radiomic analyses in clinical care, models need to account for disease prevalence and should be validated in external cohorts. Many of the presented studies solely use internal validation and do not include an external validation step and are, therefore, not ready for application in other populations. Data standardization, algorithm sharing, and other forms of multi-site collaborations may be helpful for external validation while maintaining the privacy and security of patient data.

Moreover, alternative methods of dealing with the same question involving deep learning, neural networks, or automated methods can differentiate between tumor entities and may be used successfully instead of radiomic approaches—for example, to distinguish between small-cell and non-small-cell lung cancer [136]. Current research is increasingly focusing on incorporating artificial intelligence in radiomic studies. Deep-learning methods can be used for the whole image analysis process, from image segmentation through feature extraction to final classification. Automated end-to-end machine-based learning workflows for the radiomic process may further increase the performance of radiomics and facilitate its applicability and acceptability in clinical routines. Exploring delta radiomics, a method capturing changes in radiomic features between time points, may further aid patient stratification, predict early treatment response, and consequently improve decision making in neuro-oncology [137].

## 10. Conclusions

Despite the relatively heterogeneous research on radiomic analyses, the potential of this quantitative method in patient care is high. Prospective studies on the effects of radiomics on clinical decision-making, such as image-guided diagnosis, patient stratification, treatment selection, and evaluation of early treatment response, are strongly needed.

## Supplementary Materials

The following supporting information can be downloaded at: https://www.mdpi.com/article/10.3390/cancers14030836/s1, Table S1: A Brief Overview of Radiomic Research Related to Other Less Common CNS Tumors. Reference [138] is cited in the supplementary materials.
