# Supplementary material for: Beyond Glioma: The Utility of Radiomic Analysis for Non-Glial Intracranial Tumors
Source: Cancers (Basel). 2022 Feb 7;14(3):836. doi: 10.3390/cancers14030836 (PMC8834271; doi:10.3390/cancers14030836)
Supplement: Supplementary file 1 [file cancers-14-00836-s001.zip › cancers-1512120-supplementary.pdf]

cancers

MDPI

Supplementary Materials: Beyond Glioma: The Utility of Radiomic Analysis for Non-Glial Intracranial Tumors

Darius Kalasauskas, Michael Kosterhon, Naureen Keric, Oliver Korczynski, Andrea Kronfeld, Florian Ringel, Ahmed Othman and Marc A. Brockmann

Table S1. A brief overview of radiomic research related to other less common CNS tumors.

Tumor entity	Number of patients	Imaging Sequences used	Number of features extracted	Feature selection approach	Model building approach	Validation	Accuracy	Remarks	Reference
L	195	T1CE, DWI	936	mRMR, correlation based, backward elimination	SVM, GLM, RF, MLP, human readers, convolutional NN	Internal validation, external validation	AUC up to 0.99	L vs. GBM	[58]
L	154	T1CE, DWI	1618	12 methods in total	8 machine learning classifiers	Internal validation, external validation	AUC 0.94	L vs. GBM	[62]
L	143	T1CE	36	Correlation filter, PCA, model in-built classifier	12 machine learning classifiers	Cross-validation	AUC up to 0.92	L vs. GBM	[46]
L	94	T1, T2, DWI, FLAIR, T1CE	1070	45 combinations of feature selection and model building methods		Cross-validation	AUC up to 0.98	L vs. GBM	[47]
L	289	T1CE, FLAIR, DWI	851	convolutional NN, mRMR	convolutional NN, LASSO	Cross-validation	AUC up to 0.96	L vs. GBM	[55]
L	138	T1CE		RF, LASSO, Xgboost, gradient boosting decision tree	LDA, SVM, LR	Internal validation	AUC up to 0.98	L vs. GBM	[54]
L	240	T1CE, FLAIR, DWI	851	ICC, correlation matrix, mRMR	LASSO	Cross-vendor, mixed-vendor	AUC up to 0.94	L vs. GBM	[55]
L	77	^{18}F-FDG-PET:	107	ICC, individual feature performance	individual feature performance	Cross-validation	AUC up to 0.97	L vs. GBM	[49]
L	143	T1CE, T2, DWI, FLAIR	127	mRMR, LASSO	LR, SVM, RF	External validation	AUC 0.96	L vs. GBM	[50]
L	77	T1CE, T2, FLAIR	6366	Recursive feature elimination	RF	Cross-validation	AUC 0.92	L vs. GBM	[51]
L	96	T1CE	16384	genetic algorithm	SVM	Internal validation	AUC 0.98	L vs. GBM	[52]
L	60	T1CE	67	ICC	PCA, Hierarchical clustering			L vs. GBM	[53]
L	95	T1, T1CE, T2, FLAIR	26	t-test	SVM	Cross-validation	Accuracy 97%	L vs. GBM	[56]
L	9	T1, T1CE, T2, FLAIR, ADC, DWI	198		Mann-Whitney U		AUC more than 0.97	L vs. sarcoidosis	[57]
M	18	T1CE, T2, ADC	46	Information Gain, Gini index, ReliefF, and Fast Correlation Based Filter	LR, NB, kNN, SVM, RF, NN	Cross-validation	AUC 0.96	Consistency	[92]
M	131	T1 FLAIR, T1CE FLAIR, T2	1409	The Variance Threshold method, Select K Best method, LASSO	LR, kNN; DT, SVM, RF, XGBoost	Internal validation	AUC 0.96	WHO grade	[75]
M	175	T1CE	1055	PCA, variance, correlation	RF	Internal validation	AUC 0.78*	WHO grade	[78]
M	150	T1CE	40	Distance correlation, LASSO, gradient boosting decision tree	LDA, SVM	Internal validation	AUC up to 0.93	WHO grade	[80]
M	152	ADC	156	Mutual information, Fisher coefficient and classification error probability, average correlation coefficient, recursive feature elimination	DT, conditional inference tree, decision forest	Internal validation	Accuracy up to 79.5%	WHO grade	[79]

cancers

MDPI

M	138	T1, T1CE, subtraction maps, FLAIR, ADC, T2,	12733	Mann-Whitney U	RF, SVM, XGBoost, multilayer perception	Internal validation, multicentric cohort	AUC 0.97	WHO grade	[59]
M	316	T1, T2, T1CE, ADC, SWI	3884	ICC, LASSO	RF, SMOTE	Cross-validation	AUC 0.84	WHO Grade	[82]
M	181	T1CE	2048	RF	LDA	Internal validation, multicentric cohort	AUC 0.90	WHO Grade	[76]
M	136	T1CE, ADC, DTI	90	recursive feature elimination	SVM, RF	Internal validation, cross-validation	AUC 0.86	WHO Grade	[81]
M	241	T1CE	385	Fisher discriminant analysis, Spearman correlation, t test	Fisher discriminant analysis	Cross-validation	Accuracy >91.3%	subgroup	[90]
M	108	T1CE	36		RF	Cross-validation	AUC 0.74	Brain invasion	[98]
M	454	T1CE, T2	3222	LASSO	RF	Internal validation	AUC 0.91	Brain invasion	[96]
M	1728	T1CE, T2	3190	LASSO	SVM	External validation	AUC 0.82*	Brain invasion	[95]
M	490	T1CE, T2	1227	Spearman's correlation, least absolute shrinkage, LASSO	LR	Internal validation	AUC 0.72	Bone invasion	[99]
M	25	ADC, DCE, T1, T2, FLAIR	300	LASSO		Cross-validation	AUC>0.71	Tumor vs. edema	[97]
M	136	T1CE, T2	3124	Wilcoxon rank sum test, Elastic net, recursive feature elimination	SVM	Internal validation	AUC 0.80	Postoperative edema	[94]
M	292	T1, T1CE, T2	473	concordance correlation coefficient, ICC, 16 feature selection algorithms	LR, DT, RF, SVM	Internal validation	AUC 0.92	M vs. solitary fibrous tumor	[84]
M	192	T1, T1CE, T2	396	t-test, Kruskal-Wallis, LR, LASSO, stepwise iterative method	LR	Internal validation	AUC 0.94	M vs. solitary fibrous tumor	[85]
M	67	T1CE, DWI, FLAIR	486	Boruta algorithm	SVM	Internal validation	AUC up to 0.90	M vs. solitary fibrous tumor	[86]
M	127	T1CE, T2	46	Mann–Whitney U	LR		AUC up to 0.78	M vs. craniopharyngeoma	[89]
M	303	T1CE	172	supervised false-positive avoidance methodology	RF	External validation	AUC>0.75	Prognosis	[77]
M	138	T1CE, T2, ADC	99	RF	DT	Cross-validation	Accuracy 90%	Recurrence	[91]
MB	122	T1, T1CE, FLAIR, ADC	5529	ICC, correlation matrix, RF-based	RF + clinical features	Internal validation	AUC up to 0.91*	subgroup	[70]
MB	51	T1CE, ADC	188	Student's t, MannWhitney U, LR	kNN, AdaBoost, SVM, RF	Cross-validation	AUC 0.91	MB vs. Ependymoma	[63]
MB	40	T1CE, T2, ADC	37	Feature combination	SVM	Cross-validation	Accuracy 92%	MB vs. ependymoma vs.. astrocytoma	[68]
MB	40	T1, T2	279	PCA	LDA, probabilistic NN	Cross-validation	Accuracy more then 86%	MB vs. ependymoma vs.. astrocytoma	[65]
MB	248	ADC	24		DT, NB, RF, SVM, NN	Cross-validation	AUC 0.87	Entity posterior fossa	[67]
MB	288	T1CE, T2, ADC	3087	Tree-Based Pipeline Optimization Tool, 13 feature selection algorithms	Tree-Based Pipeline Optimization Tool, 10 classifiers	Internal validation Multicentric cohort	AUC 0.92	Entity posterior fossa	[64]
MB	185	T1CE, FLAIR	10	Mann–Whitney U	LR		AUC up to 0.880	Entity posterior fossa	[69]
MB	134	T1, T2	566	ReliefF, entropy minimum descriptive length discretisation,	SVM	External validation, cross-validation	AUC up to 0.86	Tumor entity pediatric population	[66]
MB	109	T1CE, T2	590	Wilcoxon rank sum test	SVM	Cross-validation, external validation	AUC up to 0.80**	subgroup	[71]
MB	84	T1CE	385	mRMR, LASSO	LR	Internal validation, external validation	AUC 0.73*	CSF dissemination	[73]
MB	166	T1, T1CE, ADC, FLAIR, T2	5929	ICC, univariate concordance index, LASSO Cox model	Regression	Internal validation	C-index up to 0.762*	survival	[72]
BM	48	CE T1, FLAIR, APTw	110	ICC, classifier attribute evaluation filter (CfsSubsetEval) Machine Learning Classifiers (NN) Random Forest Classifiers	Machine Learning Classifiers (NN) Random Forest Classifiers	n.a.	sensitivity 81.3% specificity 81.1%	BM vs. primary brain tumors	[17]

cancers

BM	31	MRF (magnetic resonance fingerprinting)	n.a.	Spearman’s rank correlation coefficient<br>Wilcoxon rank sum test	n.a.	n.a.	AUC 0.87-0.95	BM vs. primary brain tumors	[20]
BM	166	T2 CE	265	No selection	DNN<br>7 classic machine learning classifiers	cross validation<br>external validation cohort (82 Pat., 50 GBM, 32 Met)	AUC 0.96	BM vs. primary brain tumors	[138]
BM	120	T1, T2, T1CE	321	ICC<br>Boruta algorithm	DT, SVM, neural networkNN, NB, KNN	Internal validation	Accuracy 0.64	BM vs. primary brain tumors	[11]
BM	412	T1, T2, T1CE	1303	Filter methods (T-test-score,Relief, information gain, gain ratio, Euclidean distance, F-anova (FAOV), Wilcoxon rank sum, LR, mutual information, SVM, LASSO, RF	Adaboost, kNN, MLP, DT, NB, RF, SVM	Internal validation	AUC 0.90	BM vs. primary brain tumors	[10]
BM	439	T1CE	757	neighborhood component analysis (NCA), PCA	SVM, kNN, DT<br>ensemble classifiers	Internal validation	AUC = 0.96*	BM vs. primary brain tumors	[8]
BM	100	T1CE	88	p-value	SVM<br>Naive Bayes<br>k-nearest neighbors	Cross-validation	AUC 0.90	BM vs. primary brain tumors	[12]
BM	134	T1, T1CE, T2, FLAIR	43	distance correlation,<br>RF, LASSO,<br>eXtreme gradient boosting (Xgboost),<br>Gradient Boosting Decision Tree	LDA, SVM, RF, kNN, LR, LDA, SVM, RF, kNN, Gaussian NB, LR		AUC = 0.80	BM vs. primary brain tumors	[9]
BM	120	T1, T1CE, T2, FLAIR, ADC	1070	PCA, linear combinations filter, h	12 models in total	Cross-validation	AUC up to 0.95	BM vs. primary brain tumors	[13]
BM	48	DTI	11		NB, kNN, SVM, quadratic discrimination analysis, NN	Cross-validation	AUC up ti 0.99	BM vs. primary brain tumors	[18]
BM	67	T1CE	36	Wilcoxon rank sum test	Probabilistic NN, binary decision tree	Cross-validation	Accuracy more than 93%	BM vs. primary brain tumors	[16]
BM	144	CT CE	105	Mann-Whitney-U-Test, LASSO	Binary LR, SVM, SVM + age and sex	Cross-validation	AUC 0.83	Differentiation different BM types	[32]
BM	189	T1, T1CE, FLAIR	1423	Gini impurity measures	RF	Cross-validation	AUC = 0.64-0.82	Differentiation different BM types	[29]
BM	38	T1CE	43	ANOVA, F-Test, Welchs t-Test	RF	Cross-validation	AUC = 0.61-0.94	Differentiation different BM types	[30]
BM	30	T1CE	43	Welch’s t-test	NB, kNN, multilayer perception, RF, SVM, NB, kNN, MLP, RF, SVM	Cross-validation	AUC = 0.95	Differentiation different BM types	[31]
BM	157	FLASH T1 CE (FLASH)	740	LASSO regression,	NN classifier		accuracy 80%, sensitivity 74%	response to radiation	[39]
BM	31	11C-MET PET	108	correlation matrix and point-biserial correlation coefficient	LDA		AUC 0.73	response to radiation	[40]

cancers MDPI

BM	161	T1CE	107	consensus clustering	Cox proportional hazards models, cause-specific proportional hazards models		HR 0.71*	response to radiation	[44]
BM	87	T1CE, FLAIR	440	jackknife resampling in LOPO (leave one patient out) manner resampled RF, feature importance	RF	Cross-validation	AUC 0.79*	response to radiation	[41]
BM	100	T1CE, FLAIR	3072	Pearson correlation analysis, Clutering in R-squared matrix Mann-Whitney U test in conjunction with a 50-fold sampling scheme, AUC632+ with forward feature selection, SVM classifier, ANOVA	SVM classifier + bootstrapping	Cross-validation	AUC 0.79	response to radiation	[43]
BM	89	CT	direct use of CNN	none	ensemble CNN	Internal validation	AUC 0.76-0.86	response to radiation	[42]
BM	110	T1CE, FLAIR	2,786	mRMR	Ada Boost, Gradient Boost, RF, Extra Trees	cross-validation	AUC 0.90-0.95*	response to radiation	[37]
BM	88	T1CE	21	weighted average of each radiomic feature by Met volume univariate Cox regression, false discovery rate adjustment, LASSO	multivariate analysis	Internal validation	hazard ratio 0.68	response to chemotherapy	[45]
BM	52	T1CE, T2, FLAIR, T2, DWI	438	LASSO	LR		accuracy 0.991	mutation status	[36]
BM	61	T1CE	1209	permutation RF, l0-norm minimization, infinite feature selection, a feature selection via concave minimization, mRMR, Relief, Laplacian	RF, AdaBoost, SVM, LASSO	Cross-validation, Internal validation	AUC up to 0.89	mutation status	[33]
BM	53	T1CE	195	kstest, ranksum, ttest2, and pcacov functions one-sample Kolmogorov-Smirnov test Mann-Whitney U-test or t-test PCA on features that were significantly different between the 2 groups	SVM 7 types	Cross-validation	AUC 0.78*	mutation status	[38]
BM	110	T1CE, FLAIR	2786	mRMR, SMOTE	RF, extra tree, bagging, and gradient boosting	Cross-validation	AUC 0.92-0.99*	mutation status	[34]
BM	51	T1CE, DTI	526	ICC, F score, mutual information, RFE, LASSO, tree-based method	SVM, adaptive boosting algorithm, LDA, RF	Internal validation	AUC 0.73	mutation status	[35]
BM	20	T1CE	1766	Cohen's Kappa	bagging algorithm trained with the chi-square score features set	Cross-validation	AUC 0.83	Radionecrosis vs. Progress	[24]
BM	41	11C-MET PET	42	Gini index	RF	Cross-validation	AUC 0.98	Radionecrosis vs. Progress	[28]
BM	75	T1CE	1		Wilcoxon rank sum test		AUC not provided, $p<0.05$	Radionecrosis vs. Progress	[25]
BM	66	T1CE, FLAIR	51	univariate logistic regression	IsoSVM, SVM	Cross-validation	AUC 0.81	Radionecrosis vs. Progress	[22]
BM	52	T1CE, FET-PET	42	Mann-Whitney-U test Limiting to 5 features	LR	Cross-validation	AUC up to 0.96	Radionecrosis vs. Progress	[27]

BM	47	FET-PET	62	AUCs > 0.7	n.a.		AUC up to 0.85	Radionecrosis vs. Progress	[26]
BM	87	T1, T1CE, T2, FLAIR	285	concordance correlation coefficients	RUSBoost ensemble classifier	Cross-validation	AUC 0.73	Radionecrosis vs. Progress	[23]
BM	115	T1CE	179	RFE, SVM	SVM	Cross-validation, internal validation	AUC 0.94	Radionecrosis vs. Progress	[14]
P	133	T1CE, T2	46	Mann–Whitney U	LR	Internal validation	AUC up to 0.80	P tumor vs. Rathke cleft cyst	[88]
P	235	T1CE	40	LASSO, distance correlation, RF, eXtreme gradient boosting, gradient boosting DT	LDA, SVM, RF, KNN; Adaboost, LR, Gaussian NB, DT ± gradient boosting	Internal validation	AUC more than 0.80	P vs. M vs. Craniopharyngioma vs. Rathke cleft cyst	[87]
P	112	T1CE, T1	1482	mRMR	SVM	Internal validation	AUC 0.804	Subtype	[109]
P	235	T1, T1CE, T2	2364	PCA	SVM, NB, kNN	Cross-validation	AUC up to 0.95	Subtype	[108]
P	44	T1	1021	ICC, RF, sequential forward selection	RF	Cross-validation	AUC more than 0.89	Craniopharyngeoma subtype, mutations	[111]
P	164	T1, T1CE, T2	1223	SelectKBest, LASSO, SVM with RFE	SVM	Internal validation, external validation	AUC 0.92	Craniopharyngeoma subtype	[110]
P	177	T2	107	LASSO	RF, light gradient boosting machine, extra-trees, quadratic discrimination analysis, LDA, soft voting ensemble classifier	Internal validation	AUC 0.81	Treatment response	[112]
P	57	T1, T1CE, T2	1561	SVM	LR	Cross-validation	AUC 0.96*	Treatment response	[116]
P	163	T1, T1CE, T2	1395,	SelectKBest algorithm, RFE	SVM	Internal validation	AUC 0.808	Treatment response	[117]
P	50	T1CE, T2	214	Sequential	SVM	Cross-validation	AUC 0.78	recurrence	[118]
P	27	T1CE	255	Student's t, mutual- information-based algorithm	kNN, RF, SVM, MLP, LR	Cross-validation		recurrence	[119]
P	194	T1CE, T2	194	LASSO	SVM	Internal validation	AUC 0.826	Sinus invasion	[106]
P	50	T1, T2, DCE	57	ICC, Spearman correlation, Mann-Whitney U, t-test	LR		AUC 0.957	vascular heterogeneity and aggressiveness	[107]
P	89	T2	1118	ICC, variance, correlation matrix, RFE	Extra Trees	Internal validation	AUC 0.99	consistency	[105]
P	158	T1, T1CE, T2T1CE, T1, T2	4683	Wilcoxon rank sum test, Elastic net, RFE	SVM	Internal validation, Multicentric cohort	AUC 0.81*	consistency	[103]
P	69	T1CE, T2	214	LASSO	LR	Cross-validation	AUC 0.834	Granulation pattern	[115]
P	89	T2	1128	8 feature selection algorithms	kNN	Internal validation	AUC 0.87	Proliferative index	[113]
P	138	T1, T1CE, T2,	4683	Wilcoxon rank sum test, LASSO, Elastic net, RFE	SVM	Internal validation, external validation	AUC 0.89	proliferative index	[114]
Schw	336	T2	1736	t-test, LASSOx	SVM	Internal validation	AUC up to 0.881	Pseudoprogression	[120]

Schw	85	T1CE, T1CE, T2	126	Single feature vector	SVM	Cross-validation	AUC 0.93	Prognosis	[121]
Schw	191		3918	ICC, t-test, LASSO, ANOVA	LR, RF, SVM, decision tree	Cross-validation	AUC 0.9	Blood supply	[123]
V	141	T1, T1CE, T2, FLAIR, T2 GRE	78	SVM	SVM, binary classification tree	Cross-validation	Accuracy more than 92%	Tumor entity	[15]

*including clinical predictors. Abbreviations feature selection/model building: AUC – area under the curve, DT – decision tree, GLM – generalized linear model, ICC - intraclass correlation coefficient, KNN – k nearest neighbour, LASSO - least absolute shrinkage and selection operator, LDA – linear discriminant analysis, LR – logistic regression, MLP – multilayer perception, mRMR - minimum redundancy maximum relevance, NB - Naïve Bayes, NN – neural network, PCA – principal component analysis, RF – random forest, SMOTE - synthetic minority over-sampling technique, SVM – support vector machine. Abbreviations tumor entities: BM – brain metastasis, CSF – cerebrospinal fluid, GBM – Glioblastoma, L – Lymphoma, M – meningioma, MB – Medulloblastoma, P – pituitary tumors, Schw – Schwannoma, V – various. Abbreviations imaging: ADC – apparent diffusion coefficient, APTw - Amide proton transfer weighted, C-MET – ^{11}C methionine, CE – contrast enhanced, CT – computed tomography, DCE – dynamic contrast enhanced, DTI – diffusion tensor imaging, DWI – diffusion-weighted imaging, FET - ^{18}F-flouorethyltyrosine, FLAIR – fluid-attenuated inversion recovery, FLASH – fast, low angle shot, GRE – gradient echo, PET – positron emission tomography, T1 – T1 weighted, T2 – T2 weighted, SWI – susceptibility-weighted imaging.
